# Examining four blood biomarkers for the detection of acute intracranial abnormalities following mild traumatic brain injury in older adults

**DOI:** 10.3389/fneur.2022.960741

**Published:** 2022-11-22

**Authors:** Grant L. Iverson, Mira Minkkinen, Justin E. Karr, Ksenia Berghem, Henrik Zetterberg, Kaj Blennow, Jussi P. Posti, Teemu M. Luoto

**Affiliations:** ^1^Department of Physical Medicine and Rehabilitation, Harvard Medical School, Boston, MA, United States; ^2^Department of Physical Medicine and Rehabilitation, Spaulding Rehabilitation Hospital and the Schoen Adams Research Institute at Spaulding Rehabilitation, Charlestown, MA, United States; ^3^Home Base, A Red Sox Foundation and Massachusetts General Hospital Program, Boston, MA, United States; ^4^Faculty of Medicine and Health Technology, Tampere University and Tampere University Hospital, Tampere, Finland; ^5^Department of Psychology, University of Kentucky, Lexington, KY, United States; ^6^Medical Imaging Centre, Department of Radiology, Tampere University Hospital, Tampere, Finland; ^7^Department of Psychiatry and Neurochemistry, Institute of Neuroscience and Physiology, Sahlgrenska Academy at the University of Gothenburg, Mölndal, Sweden; ^8^Clinical Neurochemistry Laboratory, Sahlgrenska University Hospital, Mölndal, Sweden; ^9^UK Dementia Research Institute at University College London, London, United Kingdom; ^10^Department of Neurodegenerative Disease, University College London Queen Square Institute of Neurology, London, United Kingdom; ^11^Hong Kong Center for Neurodegenerative Diseases, Hong Kong, Hong Kong SAR, China; ^12^Neurocenter, Department of Neurosurgery, Turku University Hospital and University of Turku, Turku, Finland; ^13^Turku Brain Injury Center, Turku University Hospital and University of Turku, Turku, Finland; ^14^Department of Neurosurgery, Tampere University Hospital and Tampere University, Tampere, Finland

**Keywords:** traumatic brain injury, biomarkers, glial fibrillary acidic protein, ubiquitin C-terminal hydrolase-L1, total tau, neurofilament light, computed tomography

## Abstract

Blood-based biomarkers have been increasingly studied for diagnostic and prognostic purposes in patients with mild traumatic brain injury (MTBI). Biomarker levels in blood have been shown to vary throughout age groups. Our aim was to study four blood biomarkers, glial fibrillary acidic protein (GFAP), ubiquitin C-terminal hydrolase-L1 (UCH-L1), neurofilament light (NF-L), and total tau (t-tau), in older adult patients with MTBI. The study sample was collected in the emergency department in Tampere University Hospital, Finland, between November 2015 and November 2016. All consecutive adult patients with head injury were eligible for inclusion. Serum samples were collected from the enrolled patients, which were frozen and later sent for biomarker analyses. Patients aged 60 years or older with MTBI, head computed tomography (CT) imaging, and available biomarker levels were eligible for this study. A total of 83 patients (mean age = 79.0, SD = 9.58, range = 60–100; 41.0% men) were included in the analysis. GFAP was the only biomarker to show statistically significant differentiation between patients with and without acute head CT abnormalities [U_(83)_ = 280, *p* < 0.001, *r* = 0.44; area under the curve (AUC) = 0.79, 95% CI = 0.67–0.91]. The median UCH-L1 values were modestly greater in the abnormal head CT group vs. normal head CT group [U _(83)_ = 492, p = 0.065, r = 0.20; AUC = 0.63, 95% CI = 0.49–0.77]. Older age was associated with biomarker levels in the normal head CT group, with the most prominent age associations being with NF-L (*r* = 0.56) and GFAP (*r* = 0.54). The results support the use of GFAP in detecting abnormal head CT findings in older adults with MTBIs. However, small sample sizes run the risk for producing non-replicable findings that may not generalize to the population and do not translate well to clinical use. Further studies should consider the potential effect of age on biomarker levels when establishing clinical cut-off values for detecting head CT abnormalities.

## Introduction

There is tremendous interest in developing blood-based biomarkers for both diagnostic and prognostic purposes for people who have sustained traumatic brain injuries (TBI) (1–4). Blood biomarkers have been studied in children (5–7) and adults (8–10) in the context of sport-related concussions (11–13) and in the emergency department (ED) setting (14, 15). There have been many studies that have employed blood-based biomarkers as a screening for trauma-related intracranial abnormalities visible on day-of-injury computed tomography (CT) (16–19). In 2018, the Food and Drug Administration (FDA) permitted the marketing of a panel of two biomarkers, glial fibrillary acidic protein (GFAP) and ubiquitin C-terminal hydrolase-L1 (UCH-L1), as a screening test for intracranial abnormalities in adults with head injuries presenting to the ED setting (20, 21). There have been multiple studies showing that GFAP is elevated in children (22, 23) and adults (14, 17–19) with structural neuroimaging evidence of TBI, and some studies have also illustrated elevations of UCH-L1 in those with macroscopic intracranial abnormalities (24, 25).

The purpose of this study was to examine the diagnostic usefulness of four serum biomarkers, from the Quanterix Simoa 4-plex assay (26), for identifying intracranial abnormalities in older adults presenting to the ED following suspected mild TBI (MTBI). Our goal was to examine the biomarkers individually—and not incorporate them into an existing clinical decision-making pathway or guideline. This 4-plex assay is used to measure four proteins: GFAP, neurofilament light (NF-L), total tau (t-tau), and UCH-L1. A description of these four biomarkers, and their temporal kinetics following TBI, is presented in Table 1. Among current trends in TBI research, there have been calls for more research focused on MTBI in older adults (27, 28), and there is evidence that biomarker results are different in older adults (29–31). Based on studies published to date, we hypothesized that, when studied in older patients, all four biomarkers would have statistically significant predictive associations with the presence of acute intracranial abnormalities on head CT, that GFAP would have the highest diagnostic accuracy, and that older patients would have higher biomarker levels.

**Table 1:** Review of the biomarkers.

Ubiquitin C-terminal hydrolase-L1: UCH-L1 is a protein primarily found in neurons in both the central and peripheral nervous system (35–38). UCH-L1 levels increase in serum (25, 39, 40) and plasma (41–43) after TBI. Serum levels become detectible within an hour and appear to reach a peak at 7–9 h post TBI (10, 25, 40). With a half-life of around 7 h, the levels decrease steadily over 48 h (10, 40, 44).
Glial fibrillary acidic protein: GFAP is a cytoskeletal intermediate filament protein almost exclusively present in astrocytes in the central nervous system (CNS) (45, 46). GFAP levels in serum (10, 39, 47, 48), and plasma (8, 41, 42, 49), increase following TBI. GFAP levels in serum begin to increase within the first hour, and appear to peak at 20 h from injury, and steadily decline over 72 h (10, 50). GFAP is reported to have a half-life of 24–48 h (44).
Tau: Tau, a microtubule-associated protein, is primarily localized in neuronal axons in the CNS (51). Levels of t-tau and its proteolytic cleavage product, c-tau, and phosphorylated tau (p-tau) increase in blood after TBI (5, 49, 52, 53), Elevated plasma levels of t-tau have been associated with repetitive MTBIs in amateur boxers (54). It has been suggested that acute plasma p-tau levels and the p-tau to t-tau ratio outperform t-tau levels in prognostication following TBI (53). Blood tau levels appear to peak 1 h following TBI and then decline over the next 12 h (52, 55). However, the levels seem to rise again between 12 and 36 h and may remain elevated up to 6 days from injury (52, 56).
Neurofilament light: NF-L is the smallest subunit of the neurofilament heteropolymer, and it is abundant in neuronal axons (57). Unlike tau, which is mostly expressed in the small unmyelinated axons (58), NF-L is found in the large-caliber myelinated axons (59). NF-L levels become elevated in blood following TBI (55, 60, 61). Serum levels increase during the first weeks from injury and the half-life has been estimated to exceed 1 week (60, 62).

## Materials and methods

### Subjects

The study sample was collected in the Tampere University Hospital ED (Tampere, Finland) between November 2015 and November 2016. The Tampere University Hospital is the only neurosurgical referral hospital in the hospital district, and the ED provides health services for a total of ~470,000 residents from 22 municipalities, both urban and rural. The Tampere University Hospital is comparable to a US level I trauma center. All adult patients aged 18 years or older, with an acute traumatic head injury, seen within 24 h of injury, were the population of interest. The minimum criteria for TBI were as follows: either blunt injury to the head or acceleration/deceleration type injury resulting in witnessed loss of consciousness, disorientation, or amnesia, and an initial Glasgow Coma Scale (GCS) score of 13–15 (32). An injury-ED admission delay of more than 24 h was an exclusion criterion. From that population, a sample of 325 patients was recruited for a prospective study (33) designed to validate the *Scandinavian Guidelines for Initial Management of Minimal, Mild and Moderate Head Injuries in Adults* (34). Of the 325 patients, 314 had blood drawn for the original study of S100B, and 225 of those samples were stored in the freezer for future use.

**Figure 1:**
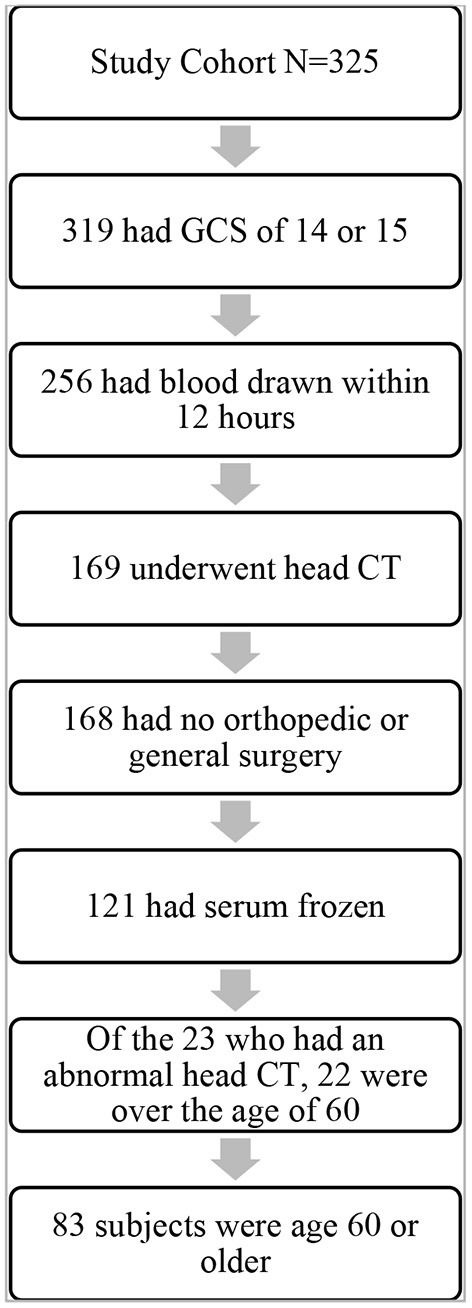
Study enrolment process. GCS, Glasgow Coma Scale; CT, computed tomography.

For the present study, the patients fulfilling the following criteria were included in the final sample: Glasgow Coma Scale (GCS) score from 14 to 15, age 60 years or over, blood sampling within 12 h or less from injury, lab results available for all four biomarkers, head CT scan performed, and no significant extracranial injury requiring surgery. Detailed case reports were obtained for each patient describing injury mechanism, symptoms following the head injury, and the findings of the physical examination performed in the ED by the on-call physician. The study enrolment process is presented in Figure 1. All enrolled patients provided an informed written consent according to the Declaration of Helsinki. The study protocol was approved by the Ethics Committee of the Pirkanmaa Hospital District, Tampere, Finland (ethical code: R15045).

### Head CT imaging

Non-contrast head CT was performed with a 64-row CT scanner (GE, Lightspeed VCT, WI, USA). The findings were systematically coded by a neuroradiologist (K.B.), blinded to the clinical characteristics of the patients, based on the National Institute of Neurological Disorders and Stroke (NINDS) Common Data Elements (CDE) (63). The following traumatic lesions were coded as acute intracranial abnormalities according to the NINDS CDEs: skull fracture, epidural hematoma, subdural hematoma, subarachnoid hemorrhage, vascular dissection, traumatic aneurysm, venous sinus injury, midline shift, cisternal compression, fourth ventricle shift/effacement, contusion, intracerebral hemorrhage, intraventricular hemorrhage, diffuse axonal injury, penetrating injuries, craniocervical junction injury, brain swelling, ischemia/infarction/hypoxic-ischemic injury. No subject had an isolated skull fracture, and an isolated skull fracture, if present, would not have met our inclusion criterion for an intracranial abnormality.

### Blood biomarkers and laboratory procedures

Venous blood samples were collected within 12 h of injury. Blood samples were centrifuged for 10 min at 10,000 rpm at room temperature. Part of the serum was analyzed at Tampere University Hospital (Tampere, Finland) as part of the hospital laboratory's on-call services for a prior study (33). The remaining serum was stored in Eppendorf tubes and immediately frozen at −70°C for future use. The blood samples were collected in Tampere between November 2015 and November 2016. Approximately 2 years later the serum was sent to the Sahlgrenska University Hospital (research laboratory) in Mölndal, Sweden for analysis. All the serum samples were transferred in 20 kilograms of dry ice from Tampere to Mölndal. The samples analyzed in Mölndal underwent one cycle of freezing and thawing. The serum samples were analyzed in March of 2018 using the Quanterix Simoa 4-plex assay on a Simoa HD-1 analyzer according to the manufacturer's instructions (Quanterix, Billerica, MA). The 4-plex assay measures four protein biomarkers in blood: GFAP, NF-L, t-tau, and UCH-L1. For GFAP, the lower limit of quantification (LLOQ) was 0.467 pg/mL, the lower limit of detection (LLOD) was 0.221 pg/mL, and the calibration range was 0–1,000 pg/mL. For NF-L, LLOQ was 0.241 pg/mL, LLOD was 0.104 pg/mL, and the calibration range was 0–500 pg/mL. For t-tau, LLOQ was 0.053 pg/mL, LLOD was 0.024 pg/mL, and the calibration range was 0–100 pg/mL. For UCH-L1, LLOQ was 5.45 pg/mL, LLOD 1.74 pg/mL, and the calibration range was 0–10 ng/mL. The mean interval in which the serum was frozen was 23.9 months (SD = 2.9, Range = 17–27). The laboratory technicians performing the analyses were blinded to the clinical data.

### Statistical analyses

The primary dependent variables were the four blood biomarkers. The distributional characteristics for these biomarkers were examined using visual inspection of histograms for each biomarker in the total sample and the subgroups and Shapiro-Wilk tests of normality. Given that nearly all biomarkers in all groups were not normally distributed, non-parametric analyses were used. Group differences were examined using Mann-Whitney *U* tests. Bivariate associations were calculated using Spearman rho coefficients. Receiver operator characteristic curves, under non-parametric assumptions, were computed using the brain imaging result as the dependent variable and the biomarker values as the predictors. The statistical analyses were conducted with the Statistical Package for Social Sciences software program (IBM SPSS Statistics for Windows, Versions 24, Armonk, NY, USA). The statistical significance level was set at *p* < 0.05.

## Results

### Patient characteristics and blood sampling

The mean age of the total sample (*n* = 83) was 79.0 years (Median = 78.0, SD = 9.58, Range = 60–100; 41.0% men). The mechanism of injury was a fall in 96.4% of the sample. All had Glasgow Coma Scale scores (GCS) of 14 or 15 in the ED (GCS 15 = 94.0%). The median time between injury and blood sampling was 3.1 h (SD = 2.2; IQR = 1.8–4.7, Range = 0.8–11.7 h). The median time between blood sampling and head CT was 0.9 h (SD = 2.4; IQR = 0.2–2.1, Range = −1.3 to 13.9 h). Intracranial abnormalities were identified in 26.5% (*n* = 22). The imaging findings were as follows: skull fracture = 2.4% (*n* = 2), epidural hematoma = 0%, extra-axial hematoma=21.7% (*n* = 18), subdural hematoma (acute) = 12.0% (*n* = 10), subdural hematoma (subacute or chronic) = 3.6% (*n* = 3), traumatic subarachnoid hemorrhage = 10.8% (*n* = 9), intraventricular hemorrhage = 1.2 (*n* = 1), midline shift (supratentorial) = 2.4% (*n* = 2), contusion = 4.8% (*n* = 4), traumatic axonal injury = 3.6% (*n* = 3). No patient had an isolated skull fracture. The injury characteristics of the sample stratified by negative and positive imaging groups (i.e., uncomplicated vs. complicated MTBI) are presented in Table 2.

**Table 2:** Injury characteristics of the samples.

	**Normal head CT (*****n*** = **61)**		**Abnormal head CT (*****n*** = **22)**	
Men, *n* (%)	25 (41)		9 (40.9)	
Women, *n* (%)	36 (59)		13 (59.1)	
	**M (SD)**	**Mdn (IQR), Range**	**M (SD)**	**Mdn (IQR), Range**
Age (years)	78.3 (9.6)	78.0 (70.0–86.0), 60–100	81.1 (9.4)	82.5 (72.8–89.0), 61–96
Time between injury and blood sampling (hours)	3.4 (2.3)	3.1 (1.7–4.5), 0.8–11.7	3.8 (2.1)	3.6 (2.2–4.8), 1.3–8.7
	**Present**		**Present**	
	***n*** **(%)**		***n*** **(%)**	
Loss of consciousness-witnessed/suspected	22 (36.1)		10 (45.5)	
Post-traumatic seizure	0 (0)		0 (0)	
Post-traumatic amnesia	18 (29.5)		13 (59.1)	
Focal neurological deficit	5 (8.2)		3 (13.6)	
Vomited 2 times or more	1 (1.6)		1 (4.5)	
Headache	28 (45.9)		10 (45.5)	
Alcohol intoxication	15 (24.6)		4 (18.2)	
Glasgow coma scale = 15	58 (95.1)		20 (90.9)	
Neurosurgery (craniotomy)	0 (0)		2 (9.1)	

CT, computed tomography; n, sample size; M, mean; SD, standard deviation; Mdn, median; IQR, interquartile range.

### Descriptive statistics and group comparisons for the biomarkers

**Figure 2:**
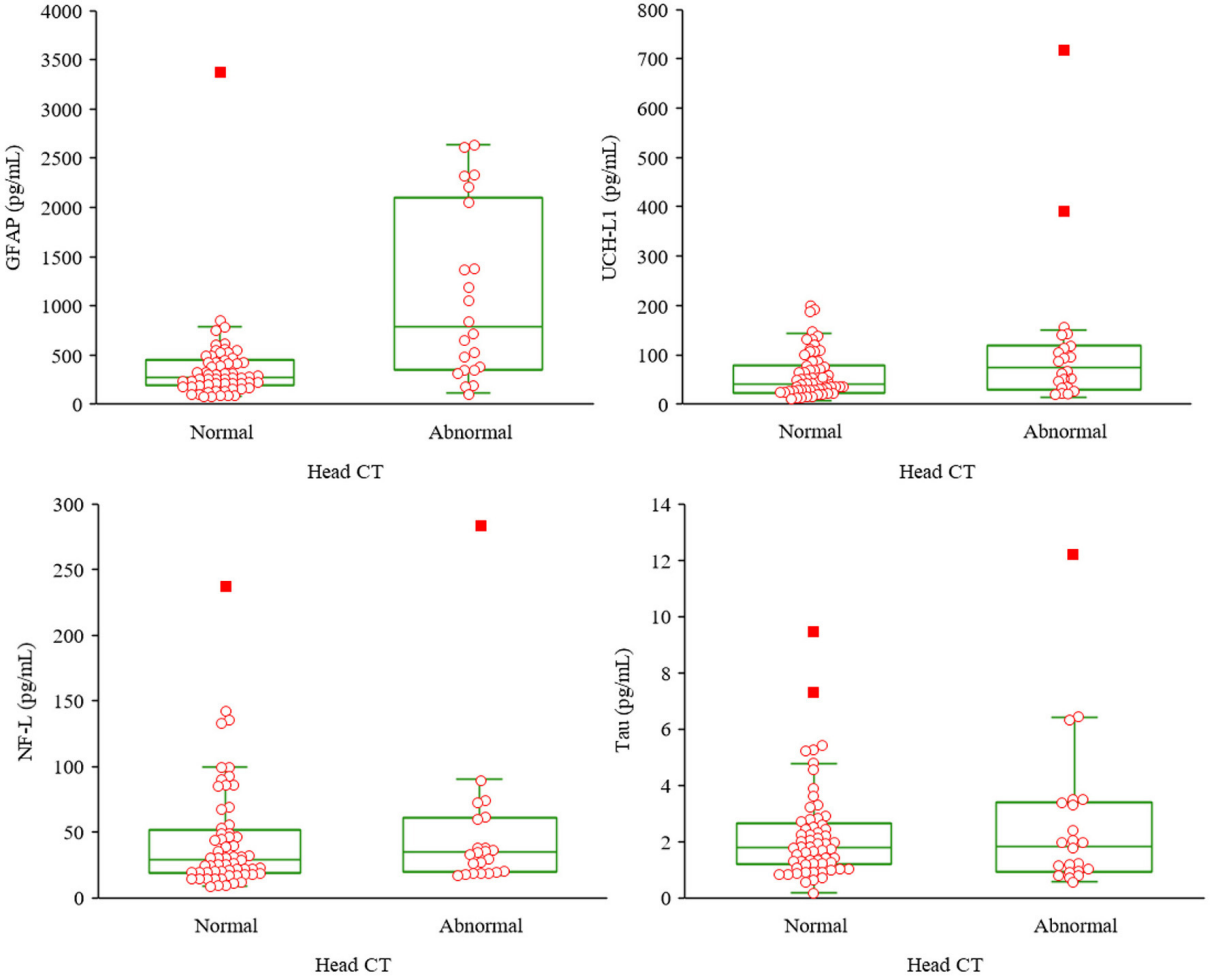
Box and whisker plots for the biomarkers by group. Upper left, glial fibrillary acidic protein; upper right, ubiquitin C-terminal hydrolase-L1; lower left, neurofilament light; lower right, total tau; CT; computed tomography. Normal head CT *n* = 61, abnormal head CT *n* = 22.

Descriptive statistics for the biomarkers, stratified by groups, are presented in Table 3 and Figure 2. Visual examination of the frequency distribution histograms and Shapiro-Wilk tests of normality revealed non-normal distributions for all biomarkers in the total sample and in the subgroups. As seen in the box and whisker plots in Figure 2, GFAP had the greatest separation between the groups, whereas the other biomarkers had similar distributional characteristics between groups. Those with abnormal CT scans had significantly greater levels of GFAP than those with normal CT scans [*U*_(83)_ = 280, *p* < 0.001, *r* = 0.44]. There were no statistically significant differences between the two groups on the other three biomarkers (NF-L, t-tau, and UCH-L1).

**Table 3:** Descriptive statistics for the biomarkers by group.

**Biomarker**	**CT finding**	***n***	**M**	**Mdn**	**SD**	**Min**.	**Max**.	**Mann-Whitney *U* Test**
GFAP	Negative	61	376.90	274.27	431.28	87.40	3,382.41	*U* = 280, *p* < 0.001, *r* = 0.44
	Positive	22	1,108.19	787.84	872.31	114.44	2,640.75	
UCH-LI	Negative	61	57.47	40.88	46.37	7.12	197.63	*U* = 492, *p* = 0.065, *r* = 0.20
	Positive	22	114.22	73.71	155.54	15.07	713.44	
NF-L	Negative	61	44.56	29.61	41.26	9.06	237.30	*U* = 612, *p* = 0.543, *r* = 0.07
	Positive	22	49.23	34.72	56.47	17.45	284.07	
t-Tau	Negative	61	2.22	1.81	1.67	0.17	9.45	*U* = 653, *p* = 0.853, *r* = −0.02
	Positive	22	2.63	1.85	2.71	0.56	12.20	

Biomarkers are reported in pg/mL. GFAP, glial fibrillary acidic protein; UCH-L1, ubiquitin C-terminal hydrolase-L1; NF-L, neurofilament light; t-Tau, total tau; CT, computed tomography; *n*, sample size; M, mean; SD, standard deviation; Mdn, median; Min., minimum; Max., maximum.

### Biomarker intercorrelations and correlations with age and time to blood sampling

**Figure 3:**
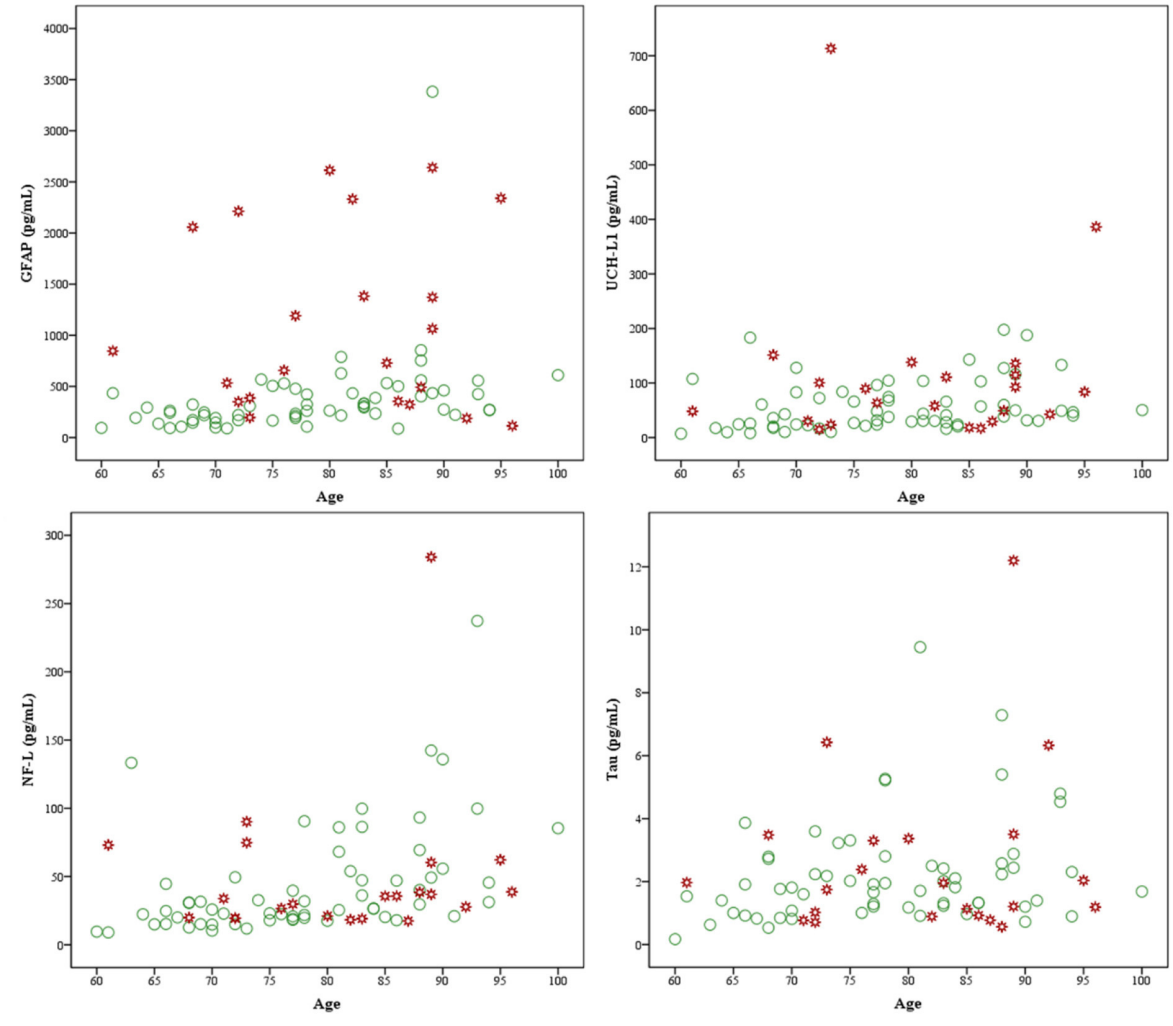
Scatterplots of biomarkers and age by group. GFAP, glial fibrillary acidic protein, upper left; UCH-L1, ubiquitin C-terminal hydrolase-L1, upper right; NF-L, neurofilament light, lower left; and tau, lower right. Markers in green are subjects with normal head computed tomography (CT) (*n* = 61) scans and markers in red are subjects with abnormal head CT scans (*n* = 22).

An intercorrelation matrix, stratified by group, is presented in Table 4. For those with a normal head CT, there were small to large correlations between age and GFAP, NF-L, t-tau, and UCH-LI. The most prominent age association was with NF-L (rho = 0.56) and GFAP (rho = 0.54), as illustrated in Table 4 and Figure 3. Due to small sample size in the abnormal head CT group, and inconsistent power across normal and abnormal head CT groupings, significance was not interpreted for the correlations. The correlations between age and the biomarkers were consistently negligible to small in the group with an abnormal head CT. The correlations between the biomarkers ranged from small to medium, apart from the large correlation between tau and UCH-L1 (rho = 0.57) in the abnormal head CT group and between GFAP and NF-L (rho = 0.53) in the normal head CT group. However, the correlation between GFAP and NF-L in the abnormal head CT group was negative (rho = −0.13). The correlations between the biomarkers and time to blood sampling varied from non-existent to weak in both groups, apart from GFAP in the abnormal head CT group (rho = 0.39).

**Table 4:** Spearman intercorrelation matrix.

		**Age**	**Time**	**GFAP**	**UCH-L1**	**NF-L**
Total (*n* = 83)	Age					
	Time to blood sampling	0.08				
	GFAP	0.37^a^	0.24^b^			
	UCH-L1	0.32^a^	−0.02	0.29^a^		
	NF-L	0.48^a^	0.08	0.37^a^	0.19	
	Tau	0.18	−0.03	0.24^b^	0.35^a^	0.28^a^
Normal CT (*n* = 61)	Age					
	Time to blood sampling	0.13				
	GFAP	0.54^a^	0.15			
	UCH-L1	0.37^a^	−0.10	0.22		
	NF-L	0.56^a^	0.06	0.53^a^	0.20	
	Tau	0.26^b^	0.03	0.36^a^	0.29^b^	0.27^b^
Abnormal CT (*n* = 22)	Age					
	Time to blood sampling	−0.13				
	GFAP	−0.06	0.39			
	UCH-L1	0.13	0.05	0.33		
	NF-L	0.23	0.12	−0.13	0.16	
	Tau	0.12	−0.10	0.23	0.57^a^	0.39

GFAP, glial fibrillary acidic protein; UCH-L1, ubiquitin C-terminal hydrolase-L1; NF-L, neurofilament light; t-Tau, total tau; CT, computed tomography.

a *p*<*0.0*1;

b *p*<*0.0*5.

### Discriminating abnormal from normal head CT scans

**Figure 4:**
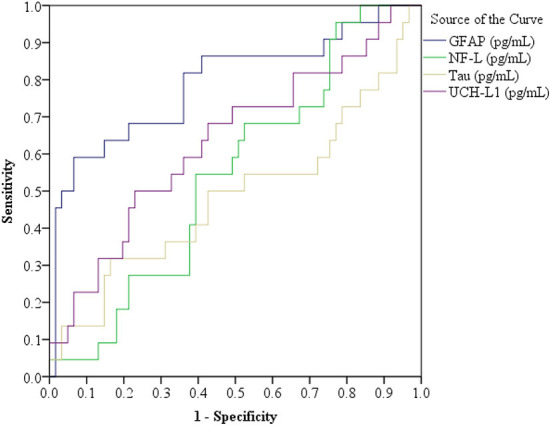
Receiver operator characteristic curves for the four biomarkers. Glial fibrillary acidic protein (GFAP), in the total sample (*N* = 83), area under the curve (AUC) = 0.791, standard error (SE) = 0.063, 95% confidence interval (CI) = 0.669–0.914; Ubiquitin C-terminal hydrolase-L1 (UCH-L1) AUC = 0.633, SE = 0.072, 95% CI = 0.493–0.774; Neurofilament light (NF-L) AUC = 0.544, SE = 0.067, 95% CI = 0.412–0.676; and t-Tau AUC = 0.487, SE = 0.079, 95% CI = 0.331–0.642. *n* = 83. A subgroup analysis was conducted for patients with Glasgow Coma Scale = 15 *(n* = 78, CT positive = 20 and normal head CT = 58), as follows: GFAP AUC = 0.776, SE = 0.068, 95% CI = 0.642–0.910; UCH-L1 AUC = 0.633, SE = 0.075, 95% CI = 0.486–0.780; NF-L AUC = 0.508, SE = 0.069, 95% CI = 0.373–0.642; and t-Tau AUC = 0.482, SE = 0.080, 95% CI = 0.325–0.639. Normal Head CT (*n* = 58): GFAP Median (Mdn) = 283.59, interquartile range (IQR) = 206.21–441.27; UCH-L1 Mdn = 41.80, IQR = 23.95–83.26; NF-L Mdn = 30.11, IQR = 19.56–54.37; and t-Tau Mdn = 1.79, IQR = 1.20–2.61. Positive Head CT (*n* = 20): GFAP Mdn = 787.84, IQR = 351.17–2174.14; UCH-L1 Mdn = 73.71, IQR = 32.83–129.61; NF-L Mdn = 32.70, IQR = 19.67–54.91; and t-Tau Mdn = 1.85, IQR = 0.95–3.35.

Receiver operator characteristic curves were computed for each biomarker. The results are presented in Figure 4. The only biomarker with a significant area under the curve was GFAP (AUC = 0.791, 95% CI = 0.669–0.914). All individuals' biomarker values are presented in the Table A1. To illustrate a possible future clinical algorithm for using two biomarkers, GFAP and UCH-L1, as a screening test for referring for head CT in older adults, we visually examined the frequency distributions and selected cutoff scores that would, in combination, identify all subjects with abnormal head CT scans. All subjects with abnormal head CT scans were correctly identified using the following algorithm: GFAP values are 323 pg/mL or greater or UCH-LI values are 42 pg/mL or greater. Applying that algorithm to those with negative head CT scans yielded the following: 36/61 (59.0%) had GFAP values of < 323 pg/mL, 31/61 (50.9%) had UCH-L1 values < 42 pg/mL, and 22/61 (36.1%) had both GFAP < 323 pg/mL and UCH-L1 < 42 pg/mL. The algorithm specificity for detecting abnormal head CT scans was 0.36 (95% CI = 0.24–0.49).

## Discussion

There is tremendous interest in whether blood biomarkers can be used within an ED clinical pathway to determine if patients should undergo head CT following a suspected or confirmed MTBI (33, 64, 65). It is recognized that biomarker values differ in older adults (30, 31), so there is a need for research focused specifically on these patients. Nearly all older adults in this study presented to the ED following head trauma sustained in a fall. It is well-established that falls are a common cause of ED visits (66, 67) and TBI in older adults (68–70). The percentage of patients with traumatic abnormalities on their head CT was 26.5%, and, although substantially higher compared to many prior MTBI studies, a high proportion of abnormal scans has been reported in other MTBI studies focusing on older adults (71, 72).

Only one of the four biomarkers, GFAP, significantly differentiated older adults with abnormal head CT scans from those with normal head CT scans (Figures 2, 4). The median values for UCH-L1 were modestly greater in the complicated MTBI group vs. the uncomplicated MTBI group (Table 3), revealing a small separation between these groups (Figure 2), whereas there was very little difference between groups on levels of NF-L and t-tau. These results align with a prior study examining the added value of incorporating GFAP and UCH-L1 into clinical decision rules for detecting CT abnormalities in MTBI, which identified improved performance using GFAP independent of UCH-L1 (73).

The AUCs in the current study were substantially lower than in a prior TBI study using the same biomarker platform (GFAP: 0.79 vs. 0.88; UCH-L1: 0.63 vs. 0.86, NF-L: 0.54 vs. 0.84, and t-tau: 0.49 vs. 0.77, respectively) (43). The prior study, however, differed methodologically in that it included patients with mild, moderate, and severe TBIs (vs. MTBI only in this study), the median age of their sample was more than 30 years younger than in the present study, and they measured the biomarkers in plasma (vs. serum in this study). By including patients with moderate and severe TBIs, the prior study had higher biomarker levels for GFAP, UCH-L1, and t-tau than the present study and this might have contributed to better differentiation of those with abnormal head CT scans. Patients with moderate and severe TBI always undergo an acute head CT (72). Therefore, in this study, we wanted to focus on patients with MTBI. We also wanted to examine older patients, because higher prevalence of intracranial abnormalities has been reported among the elderly (74) and they experience more severe consequences and slower recovery than younger patients (28).

The intercorrelations between the biomarkers ranged from very small to large, indicating their variable relationships with each other (Table 4). Older age was associated with greater levels of all the biomarkers in those with uncomplicated MTBIs (Table 4), aligning with previous studies (29, 31). In contrast, for those with abnormal head CT scans, there was not an association between age and biomarker levels (Table 4; Figure 3). We found a large correlation between GFAP and NF-L (*r* = 0.53) in patients with normal head CT scans, while in patients with abnormal head CT scans, a similar correlation was found between UCH-L1 and t-tau (*r* = 0.57). The prior study with the same biomarker platform found the strongest correlation between NF-L and UCH-L1 (*r* = 0.71) and the weakest correlation between GFAP and t-tau (*r* = 0.06) (43). In their study cohort, the median age was 36 years lower and all TBI severities were included. While the neurobiology underlying this difference in results is unclear, the intercorrelations of biomarker concentrations are possibly also affected by age, sampling time point, extracranial injuries, and the severity of TBI.

Given that GFAP and UCH-L1 have been FDA-approved to screen for abnormal head CT scans following TBI in adults, we provided all individual subjects' biomarker values in the Table A1. Some authors have expressed concern that past researchers have not attempted to develop clinical cutoff scores or develop clinical algorithms for using blood biomarkers in the ED setting (75, 76). Therefore, we illustrated one example of an algorithm that can be used within 12 h of injury, optimized for the data presented in the Table A1, that yielded 100% sensitivity for identifying abnormal head CT scans. The algorithm produced 39 false positive and 22 true negative results, reflecting a specificity of 36.1% within the sample. Although the algorithm was developed using biomarker levels of older adults, the specificity of the algorithm matches that of the FDA-approved biomarker kit using the same biomarker combination (specificity 0.367) (20). A summary of the individual biomarkers follows.

### GFAP

We found that GFAP was the only biomarker that significantly differentiated older adults with abnormal head CT scans with the AUC of 0.791. The use of this biomarker in detecting CT abnormalities in TBI patients has considerable support in the literature. A living systematic review summarized the existing literature on GFAP as a screening test for abnormal head CT findings (77). The sensitivities for predicting abnormal head CT findings were between 67 and 100% and the specificities between 0 and 89% (77). Different assays have been used in detecting GFAP levels, with various ELISA assays being the most often used (10, 14, 39, 41, 47, 48, 50, 78). Others have used assays by Randox Biochip (42), digital array from Quanterix™ (49), and the Quanterix Simoa 4-plex (8, 17, 43). GFAP is included in the FDA approved the Banyan BTI™ for clinical use (21). A study raised concern in the ability of GFAP to differentiate between patients with MTBI vs. orthopedic injury (79), which may be in part a result of GFAP not being elevated in some cases of uncomplicated MTBI (16, 80). However, it has been reported that serum GFAP levels may differentiate between patients with moderate to severe TBI and orthopedic controls 30 days after the injury (81). GFAP has been shown to be less accurate in discriminating complicated and uncomplicated MTBI patients in older vs. younger adults (30). The positive correlation between age and GFAP in our sample is consistent with a study (29) that reported greater GFAP concentrations in older patients with MTBIs.

### UCH-L1

In the current study, we found that UCH-L1 did not statistically significantly differentiate older adults with abnormal head CT scans. Those with abnormal CT scans had modestly higher UCH-L1 levels, but the effect size was small. Prior studies illustrate that UCH-L1 levels differentiate, to some degree, patients with and without traumatic intracranial CT findings in isolated TBI, and in people with TBI and multitrauma (17, 18, 20, 24, 25). A living systematic review summarized the existing literature on serum and plasma UCH-L1 for predicting acute traumatic head CT findings. The AUC for serum was 0.77 (95% CI: 0.70–0.83, 347 cases, 179 controls) and for plasma was 0.67 (95% CI: 0.58–0.76, 595 cases, 256 control) (77). Several different assays for UCH-L1 have been used in TBI studies. Most studies have used ELISA, either custom made (25, 39, 40, 82–84) or commercially available by Banyan Biomarkers Inc. (6, 10, 20, 24, 41, 50, 85). Other studies have used Randox Biochip (42, 79, 86), the Quanterix Simoa 4-plex (17, 43), or electrochemiluminescence immunosassays (87). They differ in analysis techniques resulting in different limits of detection and quantification. UCH-L1 is included in the FDA approved the Banyan BTI™ for clinical use along with GFAP (21). Although considered brain-specific, a study detected elevated UCH-L1 serum levels in patients with orthopedic trauma compared to the healthy controls (25). Also, one study revealed that UCH-L1 levels did not distinguish patients with orthopedic injury without head trauma from patients with uncomplicated MTBI (79). In our study, there were modest differences in the median values of UCH-L1 (*r* = 0.20) between groups (Table 3), and we were statistically underpowered to detect a difference of this small magnitude. A recent study (29) showed the Banyan BTI™ to be 100% sensitive in detecting CT abnormalities in older patients but showed a stepwise reduction in specificity with age. The specificity for all patients over 65 years old was 13%. In our study, UCH-L1 was useful in an algorithm combined with GFAP designed to reduce the number of CT scans in the CT negative group while detecting all abnormalities in the CT positive group. The specificity of the algorithm was 36%.

### Tau

In the current study, we found that t-tau did not statistically significantly differentiate older adults with abnormal head CT scans. There are discrepancies in other study results assessing the ability of tau to discriminate patients with and without head CT findings following milder spectrum brain injury (9, 17, 88). One concern expressed previously was that studies showing no significant differences in serum tau levels based on head CT findings involved an assay with a relatively high lower limit of detection, possibly obtaining negative findings due to less sensitivity of the assay (54). Studies using more sensitive technology have showed significant increases in blood tau levels in patients with MTBIs with abnormal findings on head CT (17, 43, 49). In addition, it should be noted, that there are studies suggesting that blood tau levels are affected by factors such as age in cognitively normal individuals (89) and presence of Alzheimer's disease (90, 91). In the current study, we used one of the more sensitive technologies for measuring t-tau with a lower limit of detection but found no group differences based on CT findings.

### NF-L

In our study, NF-L did not statistically significantly differentiate older adults with abnormal head CT scans. In some studies, NF-L levels in blood discriminate patients with and without traumatic head CT findings (17, 43, 55). Elevated levels have been linked to diffuse axonal injury (92). However, due to its kinetically slow increase profile in blood, some suggest it might be more suitable for sampling during subacute and chronic stages of TBI for outcome prediction purposes (62). Also, blood NF-L levels have been reported to be higher in neurological conditions such as Alzheimer's disease (93, 94), multiple sclerosis (95), and Huntington's disease (96). A strong association between older age and increased NF-L levels has been reported both in MTBI patients and orthopedically injured trauma controls (31). Specifically, there was a high correlation between age and NF-L levels in a sample of adults with MTBIs and normal day-of-injury neuroimaging (*r* = 0.80), and within subgroups of patients who did (*r* = 0.068) and did not (*r* = 0.76) have pre-injury neurological diseases (31).

## Limitations

This study was limited in its sample size, and especially, in the small number of patients with complicated MTBI. The sample was underpowered to detect smaller effects, but the overall differences in biomarker levels were quite modest with the exception of GFAP.

Small sample sizes run the risk for producing non-replicable findings that may not generalize to the population and do not translate well to clinical use. The results of this study require replication both in a larger sample of older adults and in younger patients. Moreover, GFAP levels might reflect, in part, aging-related inflammatory processes affecting astrocytes (97), higher levels of GFAP are associated with both aging and the development of dementia (98), and one study suggests that GFAP might be less sensitive to traumatic intracranial abnormalities in older adults compared to younger adults (30). The current approach of using the 4-plex to detect abnormal head CT should be replicated in diverse samples by future researchers to evaluate potential clinical usefulness.

Extracranial injuries were only grossly assessed based on the need for surgery other than neurosurgery, and more minor orthopedic injuries, such as conservatively treated fractures, were not noted. Therefore, it is possible that the biomarker concentrations are affected by these injuries. Our data is not suitable for analyzing the possible effect of extracranial injuries on biomarker concentrations.

Measuring biomarkers in cerebrospinal fluid (CSF) might yield different results. However, there are practical (e.g., time and cost), clinical (e.g., risks and possible need to discontinue anticoagulants), and ethical (e.g., suitable less invasive methods are available) challenges associated with measuring biomarkers in CSF.

Finally, our blood samples were stored in low temperature for ~2 years before the biomarker analysis. Although freezing blood samples in low temperatures has been shown to preserve proteins for years to decades (99), it is uncertain if the biomarker concentrations may have been affected by the storage period.

## Conclusions

This study set out to determine if GFAP, UCH-L1, NF-L, and tau differentiated between older adult patients with and without traumatic intracranial abnormalities due to MTBI. GFAP was the only biomarker that significantly differentiated older adults with abnormal head CT scans from those with normal scans. The findings support the use of GFAP as a biomarker for detecting CT-positive intracranial abnormalities in older adults with MTBI. Further studies should consider the potential effect of age on biomarker levels when establishing clinical cut-off values for detecting head CT abnormalities.

## Data availability statement

The original contributions presented in the study are included in the article/Supplementary material, further inquiries can be directed to the corresponding author.

## Ethics statement

The studies involving human participants were reviewed and approved by Ethics Committee of the Pirkanmaa Hospital District, Tampere, Finland (ethical code: R15045). The patients/participants provided their written informed consent to participate in this study.

## Author contributions

GI conceptualized the study, assisted with the literature review, conceptualized the statistical analyses, conducted the analyses, and wrote portions of the manuscript. MM assisted with conceptualizing the study, conducted the literature review, and wrote portions of the manuscript. JK assisted with conceptualizing the statistical analyses. KB reviewed and coded all the head CT scans. HZ and KajB conducted all the blood biomarker analyses, provided the results in a database, and wrote portions of the manuscript. JP assisted with conceptualizing the study and assisted with the literature review. TL assisted with conceptualizing the study, served as the overall study PI for the parent study and this research program, managed all regulatory requirements, and supervised the conduct of all aspects of data collection and database development. All authors critically reviewed and edited drafts of the manuscript and approved the final version for submission.

## Funding

The study was financially supported by the Finnish State Research Funding, and the Finnish Medical Society Duodecim. TL and JP have received funding from Government's Special Financial Transfer tied to academic research in Health Sciences (Finland). JP has received funding from the Academy of Finland (#17379), Emil Aaltonen Foundation sr and the Maire Taponen Foundation. KajB acknowledges funding from the Torsten Söderberg Foundation, the Swedish Research Council, and the Swedish Brain Foundation. HZ is a Wallenberg Scholar supported by grants from the Swedish Research Council (#2018-02532), the European Research Council (#681712 and #101053962), Swedish State Support for Clinical Research (#ALFGBG-71320), the Alzheimer Drug Discovery Foundation (ADDF), USA (#201809-2016862), the AD Strategic Fund and the Alzheimer's Association (#ADSF-21-831376-C, #ADSF-21-831381-C and #ADSF-21-831377-C), the Olav Thon Foundation, the Erling-Persson Family Foundation, Stiftelsen för Gamla Tjänarinnor, Hjärnfonden, Sweden (#FO2019-0228), the European Union's Horizon 2020 research and innovation programme under the Marie Skłodowska-Curie grant agreement No 860197 (MIRIADE), the European Union Joint Programme—Neurodegenerative Disease Research (JPND2021-00694), and the UK Dementia Research Institute at UCL (UKDRI-1003). GI acknowledges unrestricted philanthropic support from the Mooney-Reed Charitable Foundation, ImPACT Applications, Inc., the Heinz Family Foundation, and the Schoen Adams Research Institute at Spaulding Rehabilitation. The above entities were not involved in the study design, collection, analysis, interpretation of data, the writing of this article, or the decision to submit it for publication.

## Conflict of interest

Author GI serves as a scientific advisor for NanoDX^®^, Sway Operations, LLC, and Highmark, Inc. He has a clinical and consulting practice in forensic neuropsychology, including expert testimony, involving individuals who have sustained mild TBIs. He has received research funding from several test publishing companies, including ImPACT Applications, Inc., CNS Vital Signs, and Psychological Assessment Resources (PAR, Inc.). He has received research funding as a principal investigator from the National Football League, and subcontract grant funding as a collaborator from the Harvard Integrated Program to Protect and Improve the Health of National Football League Players Association Members. Author JP has received a speaker's fee Finnish Medical Association. Author HZ has served at scientific advisory boards and/or as a consultant for Abbvie, Alector, Annexon, Apellis, Artery Therapeutics, AZTherapies, CogRx, Denali, Eisai, Nervgen, Novo Nordisk, Pinteon Therapeutics, Red Abbey Labs, Passage Bio, Roche, Samumed, Siemens Healthineers, Triplet Therapeutics, and Wave, has given lectures in symposia sponsored by Cellectricon, Fujirebio, Alzecure, Biogen, and Roche, and is a co-founder of Brain Biomarker Solutions in Gothenburg AB (BBS), which is a part of the GU Ventures Incubator Program. Author KajB has served as a consultant or at advisory boards for Alzheon, BioArctic, Biogen, Eli Lilly, Fujirebio Europe, IBL International, Merck, Novartis, Pfizer, and Roche Diagnostics, and is a co-founder of Brain Biomarker Solutions in Gothenburg AB, a GU Venture-based platform company at the University of Gothenburg. The remaining authors declare that the research was conducted in the absence of any commercial or financial relationships that could be construed as a potential conflict of interest.

## Publisher's note

All claims expressed in this article are solely those of the authors and do not necessarily represent those of their affiliated organizations, or those of the publisher, the editors and the reviewers. Any product that may be evaluated in this article, or claim that may be made by its manufacturer, is not guaranteed or endorsed by the publisher.

## Figures and Tables

**Table A1:** Individual subjects' biomarker values (pg/mL) sorted by GFAP and by group.

**Normal head CT**						**Normal head CT**						**Abnormal head CT**					
**ID**	**Age**	**GFAP**	**UCH-L1**	**NF-L**	**t-tau**	**ID**	**Age**	**GFAP**	**UCH-LI**	**NF-L**	**t-tau**	**ID**	**Age**	**GFAP**	**UCH-L1**	**NF-L**	**t-tau**
339	86.0	87.404	**102.990**	18.001	1.321	184	64.0	292.916	9.860	22.368	1.399	355	96.0	114.444	**386.304**	38.783	1.189
287	71.0	90.265	22.706	22.874	1.598	138	83.0	294.218	27.986	86.380	1.312	360	92.0	188.588	**42.706**	27.766	6.328
204	66.0	92.736	8.237	44.632	1.909	095	83.0	306.735	40.877	36.157	2.416	271	73.0	195.592	**713.444**	90.163	6.424
203	60.0	94.557	7.119	9.632	0.171	225	73.0	308.590	10.376	11.916	2.178	228	87.0	**323.110**	29.540	17.451	0.774
144	70.0	98.566	**127.998**	10.392	0.814	286	68.0	322.340	17.762	30.608	2.720	351	72.0	**349.994**	15.073	19.463	0.708
149	67.0	105.283	**60.608**	20.051	0.826	245	78.0	**323.188**	37.743	21.813	5.261	295	86.0	**354.694**	17.039	35.672	0.924
224	78.0	106.699	**74.218**	31.925	1.948	135	83.0	**330.417**	16.049	47.272	1.234	277	73.0	**386.933**	23.281	74.804	1.745
214	65.0	135.817	24.390	14.976	1.004	108	83.0	**332.453**	**65.819**	99.755	2.013	230	88.0	**492.604**	**49.188**	38.454	0.563
132	70.0	145.567	**83.035**	25.808	1.811	231	84.0	**387.479**	23.859	26.716	1.820	107	71.0	**533.145**	30.386	33.862	0.767
343	68.0	146.852	35.917	12.708	2.789	153	88.0	**403.082**	**127.456**	40.205	7.286	120	76.0	**657.108**	**89.672**	26.612	2.385
179	75.0	166.290	**65.977**	17.919	2.019	159	78.0	**422.222**	**104.450**	90.539	5.216	175	85.0	**729.044**	18.268	35.572	1.133
357	72.0	169.771	**72.476**	15.103	3.594	192	93.0	**424.888**	**133.234**	99.772	4.534	189	61.0	**846.644**	**48.201**	73.089	1.968
262	68.0	170.991	20.162	31.041	0.533	161	82.0	**433.279**	30.515	53.901	2.500	267	89.0	**1,064.121**	**114.594**	284.068	12.200
279	70.0	190.954	24.056	14.843	1.074	353	61.0	**434.286**	**107.582**	9.055	1.531	232	77.0	**1,191.149**	**63.553**	29.823	3.300
094	77.0	192.155	**47.981**	20.806	1.661	263	89.0	**434.825**	**49.901**	49.223	2.439	157	89.0	**1,372.245**	**135.899**	36.954	1.208
331	63.0	193.154	17.360	133.355	0.627	152	90.0	**460.618**	**187.730**	135.883	0.721	235	83.0	**1,382.719**	**110.735**	19.128	1.953
260	77.0	210.562	31.717	18.851	1.912	163	77.0	**477.162**	23.982	18.281	1.287	302	68.0	**2,057.879**	**151.345**	19.913	3.481
257	81.0	215.777	**103.772**	25.452	1.706	278	86.0	**501.701**	**57.073**	46.989	1.329	215	72.0	**2,212.887**	**100.462**	19.594	1.021
268	69.0	218.441	10.236	15.132	0.846	281	75.0	**504.937**	26.802	23.078	3.306	321	82.0	**2,331.838**	**58.248**	18.326	0.890
205	72.0	219.909	16.674	49.444	2.233	206	76.0	**529.588**	21.441	22.439	1.004	246	95.0	**2,340.981**	**83.872**	62.243	2.036
325	91.0	221.865	30.721	20.950	1.395	140	85.0	**532.299**	**143.043**	20.353	0.969	198	80.0	**2,613.821**	**138.220**	21.106	3.366
172	77.0	233.167	**96.265**	39.659	1.207	273	93.0	**557.109**	**49.105**	237.303	4.794	363	89.0	**2,640.746**	**92.724**	60.286	3.503
258	84.0	235.187	20.315	26.332	2.104	237	88.0	**560.016**	38.680	29.610	5.401						
207	66.0	243.461	**183.169**	24.739	3.866	187	74.0	**567.810**	**83.933**	32.702	3.226						
297	69.0	245.478	**42.728**	31.575	1.766	104	100.0	**610.401**	**50.455**	85.437	1.684						
212	78.0	260.781	**67.890**	19.801	2.802	310	81.0	**627.396**	30.670	86.047	0.911						
109	66.0	262.556	25.743	15.252	0.919	283	88.0	**753.972**	**59.750**	69.423	2.232						
253	80.0	263.371	29.421	17.566	1.176	199	81.0	**788.310**	**43.798**	68.159	9.448						
196	94.0	267.663	40.316	45.553	2.305	142	88.0	**853.919**	**197.634**	93.206	2.577						
177	94.0	272.902	**46.483**	31.277	0.894	315	89.0	**3,382.409**	**116.109**	142.353	2.880						
328	90.0	274.270	31.590	55.782	1.203												

GFAP, glial fibrillary acidic protein; UCH-L1, ubiquitin C-terminal hydrolase-L1; NF-L, neurofilament light; t-Tau, total tau; CT, computed tomography; Values in bold are above the study-specific cutoff scores. Optimized, Study-Specific Algorithm: If GFAP value is 323 or greater or UCH-LI value is 42 or greater then positive head CT sensitivity = 100%. Normal head CT *n* = 61, abnormal head CT *n* = 22.
