# Supplementary material for: Examining four blood biomarkers for the detection of acute intracranial abnormalities following mild traumatic brain injury in older adults
Source: Front Neurol. 2022 Nov 22;13:960741. doi: 10.3389/fneur.2022.960741 (PMC9723459; doi:10.3389/fneur.2022.960741)
Supplement: Supplementary file 1 [file Data_Sheet_1.PDF]

**Online Supplement**

# Examining Four Blood Biomarkers for the Detection of Acute Intracranial Abnormalities Following Mild Traumatic Brain Injury in Older Adults

Grant L. Iverson[1-3], Mira Minkkinen[4], Justin E. Karr[5], Ksenia Berghem[6], Henrik Zetterberg[7,8,9,10,11], Kaj Blennow[7,8], Jussi P. Posti[12,13], Teemu M. Luoto[14]*

1. Department of Physical Medicine and Rehabilitation, Harvard Medical School, Boston, MA, USA.
2. Department of Physical Medicine and Rehabilitation, Spaulding Rehabilitation Hospital and the Schoen Adams Research Institute at Spaulding Rehabilitation, Charlestown, MA, USA.
3. Home Base, A Red Sox Foundation and Massachusetts General Hospital Program, Charlestown, MA, USA.
4. Faculty of Medicine and Health Technology, Tampere University, Tampere, Finland.
5. Department of Psychology, University of Kentucky, Lexington, KY, USA.
6. Medical Imaging Centre, Department of Radiology, Tampere University Hospital, Tampere, Finland.
7. Institute of Neuroscience and Physiology, Department of Psychiatry and Neurochemistry, the Sahlgrenska Academy at the University of Gothenburg, Mölndal, Sweden.
8. Clinical Neurochemistry Laboratory, Sahlgrenska University Hospital, Mölndal, Sweden.
9. UK Dementia Research Institute at University College London, London, United Kingdom.
10. Department of Neurodegenerative Disease, University College London Institute of Neurology, Queen Square, London, United Kingdom.
11. Hong Kong Center for Neurodegenerative Diseases, Hong Kong, China.
12. Neurocenter, Department of Neurosurgery, Turku University Hospital, and University of Turku, Turku, Finland.
13. Turku Brain Injury Center, Turku University Hospital, and University of Turku, Turku, Finland.
14. Department of Neurosurgery, Tampere University Hospital and Tampere University, Tampere, Finland.

| Brain Imaging Result | | | GFAP (pg/mL) | UCH-L1 (pg/mL) | NF-L (pg/mL) | Tau (pg/mL) |
|---|---|---|---|---|---|---|
| Normal | N | Valid | 61 | 61 | 61 | 61 |
| | | Missing | 0 | 0 | 0 | 0 |
| | Mean | | 376.90190 | 57.47449 | 44.56353 | 2.22394 |
| | Median | | 274.27034 | 40.87659 | 29.60972 | 1.81073 |
| | Std. Deviation | | 431.278908 | 46.367746 | 41.261301 | 1.666803 |
| | Skewness | | 5.835 | 1.386 | 2.394 | 2.159 |
| | Std. Error of Skewness | | .306 | .306 | .306 | .306 |
| | Kurtosis | | 40.384 | 1.429 | 7.380 | 5.931 |
| | Std. Error of Kurtosis | | .604 | .604 | .604 | .604 |
| | Minimum | | 87.404 | 7.119 | 9.055 | .171 |
| | Maximum | | 3382.409 | 197.634 | 237.303 | 9.448 |
| | Percentiles | 25 | 192.65426 | 24.01897 | 19.32586 | 1.18961 |
| | | 50 | 274.27034 | 40.87659 | 29.60972 | 1.81073 |
| | | 75 | 447.72125 | 78.62643 | 51.67228 | 2.64838 |
| Abnormal | N | Valid | 22 | 22 | 22 | 22 |
| | | Missing | 0 | 0 | 0 | 0 |
| | Mean | | 1108.19498 | 114.21624 | 49.23325 | 2.63026 |
| | Median | | 787.84394 | 73.71261 | 34.71695 | 1.84893 |
| | Std. Deviation | | 872.309275 | 155.542353 | 56.465960 | 2.707729 |
| | Skewness | | .642 | 3.193 | 3.731 | 2.453 |
| | Std. Error of Skewness | | .491 | .491 | .491 | .491 |
| | Kurtosis | | -1.124 | 11.136 | 15.550 | 6.989 |
| | Std. Error of Kurtosis | | .953 | .953 | .953 | .953 |
| | Minimum | | 114.444 | 15.073 | 17.451 | .563 |
| | Maximum | | 2640.746 | 713.444 | 284.068 | 12.200 |
| | Percentiles | 25 | 353.51860 | 30.17497 | 19.83301 | .91570 |
| | | 50 | 787.84394 | 73.71261 | 34.71695 | 1.84893 |
| | | 75 | 2096.63146 | 119.92074 | 60.77546 | 3.39462 |

| GFAP (pg/mL) | | | | | | | |
|---|---|---|---|---|---|---|---|
| Brain Imaging Result | | | Frequency | Percent | Valid Percent | Cumulative Percent |
| Normal | Valid | 87.404 | 1 | 1.6 | 1.6 | 1.6 |
| | | 90.265 | 1 | 1.6 | 1.6 | 3.3 |
| | | 92.736 | 1 | 1.6 | 1.6 | 4.9 |
| | | 94.557 | 1 | 1.6 | 1.6 | 6.6 |
| | | 98.566 | 1 | 1.6 | 1.6 | 8.2 |
| | | 105.283 | 1 | 1.6 | 1.6 | 9.8 |
| | | 106.699 | 1 | 1.6 | 1.6 | 11.5 |
| | | 135.817 | 1 | 1.6 | 1.6 | 13.1 |
| | | 145.567 | 1 | 1.6 | 1.6 | 14.8 |
| | | 146.852 | 1 | 1.6 | 1.6 | 16.4 |
| | | 166.290 | 1 | 1.6 | 1.6 | 18.0 |
| | | 169.771 | 1 | 1.6 | 1.6 | 19.7 |
| | | 170.991 | 1 | 1.6 | 1.6 | 21.3 |
| | | 190.954 | 1 | 1.6 | 1.6 | 23.0 |
| | | 192.155 | 1 | 1.6 | 1.6 | 24.6 |
| | | 193.154 | 1 | 1.6 | 1.6 | 26.2 |
| | | 210.562 | 1 | 1.6 | 1.6 | 27.9 |
| | | 215.777 | 1 | 1.6 | 1.6 | 29.5 |
| | | 218.441 | 1 | 1.6 | 1.6 | 31.1 |
| | | 219.909 | 1 | 1.6 | 1.6 | 32.8 |
| | | 221.865 | 1 | 1.6 | 1.6 | 34.4 |
| | | 233.167 | 1 | 1.6 | 1.6 | 36.1 |
| | | 235.187 | 1 | 1.6 | 1.6 | 37.7 |
| | | 243.461 | 1 | 1.6 | 1.6 | 39.3 |
| | | 245.478 | 1 | 1.6 | 1.6 | 41.0 |
| | | 260.781 | 1 | 1.6 | 1.6 | 42.6 |
| | | 262.556 | 1 | 1.6 | 1.6 | 44.3 |
| | | 263.371 | 1 | 1.6 | 1.6 | 45.9 |
| | | 267.663 | 1 | 1.6 | 1.6 | 47.5 |
| | | 272.902 | 1 | 1.6 | 1.6 | 49.2 |
| | | 274.270 | 1 | 1.6 | 1.6 | 50.8 |
| | | 292.916 | 1 | 1.6 | 1.6 | 52.5 |
| | | 294.218 | 1 | 1.6 | 1.6 | 54.1 |
| | | 306.735 | 1 | 1.6 | 1.6 | 55.7 |
| | | 308.590 | 1 | 1.6 | 1.6 | 57.4 |
| | | 322.340 | 1 | 1.6 | 1.6 | 59.0 |
| | | 323.188 | 1 | 1.6 | 1.6 | 60.7 |
| | | 330.417 | 1 | 1.6 | 1.6 | 62.3 |
| | | 332.453 | 1 | 1.6 | 1.6 | 63.9 |
| | | 387.479 | 1 | 1.6 | 1.6 | 65.6 |
| | | 403.082 | 1 | 1.6 | 1.6 | 67.2 |
| | | 422.222 | 1 | 1.6 | 1.6 | 68.9 |
| | | 424.888 | 1 | 1.6 | 1.6 | 70.5 |
| | | 433.279 | 1 | 1.6 | 1.6 | 72.1 |
| | | 434.286 | 1 | 1.6 | 1.6 | 73.8 |
| | | 434.825 | 1 | 1.6 | 1.6 | 75.4 |

| GFAP (pg/mL) | | | | | | |
|---|---|---|---|---|---|---|
| Brain Imaging Result | | | Frequency | Percent | Valid Percent | Cumulative Percent |
| | | 460.618 | 1 | 1.6 | 1.6 | 77.0 |
| | | 477.162 | 1 | 1.6 | 1.6 | 78.7 |
| | | 501.701 | 1 | 1.6 | 1.6 | 80.3 |
| | | 504.937 | 1 | 1.6 | 1.6 | 82.0 |
| | | 529.588 | 1 | 1.6 | 1.6 | 83.6 |
| | | 532.299 | 1 | 1.6 | 1.6 | 85.2 |
| | | 557.109 | 1 | 1.6 | 1.6 | 86.9 |
| | | 560.016 | 1 | 1.6 | 1.6 | 88.5 |
| | | 567.810 | 1 | 1.6 | 1.6 | 90.2 |
| | | 610.401 | 1 | 1.6 | 1.6 | 91.8 |
| | | 627.396 | 1 | 1.6 | 1.6 | 93.4 |
| | | 753.972 | 1 | 1.6 | 1.6 | 95.1 |
| | | 788.310 | 1 | 1.6 | 1.6 | 96.7 |
| | | 853.919 | 1 | 1.6 | 1.6 | 98.4 |
| | | 3382.409 | 1 | 1.6 | 1.6 | 100.0 |
| | | Total | 61 | 100.0 | 100.0 | |
| Abnormal | Valid | 114.444 | 1 | 4.5 | 4.5 | 4.5 |
| | | 188.588 | 1 | 4.5 | 4.5 | 9.1 |
| | | 195.592 | 1 | 4.5 | 4.5 | 13.6 |
| | | 323.110 | 1 | 4.5 | 4.5 | 18.2 |
| | | 349.994 | 1 | 4.5 | 4.5 | 22.7 |
| | | 354.694 | 1 | 4.5 | 4.5 | 27.3 |
| | | 386.933 | 1 | 4.5 | 4.5 | 31.8 |
| | | 492.604 | 1 | 4.5 | 4.5 | 36.4 |
| | | 533.145 | 1 | 4.5 | 4.5 | 40.9 |
| | | 657.108 | 1 | 4.5 | 4.5 | 45.5 |
| | | 729.044 | 1 | 4.5 | 4.5 | 50.0 |
| | | 846.644 | 1 | 4.5 | 4.5 | 54.5 |
| | | 1064.121 | 1 | 4.5 | 4.5 | 59.1 |
| | | 1191.149 | 1 | 4.5 | 4.5 | 63.6 |
| | | 1372.245 | 1 | 4.5 | 4.5 | 68.2 |
| | | 1382.719 | 1 | 4.5 | 4.5 | 72.7 |
| | | 2057.879 | 1 | 4.5 | 4.5 | 77.3 |
| | | 2212.887 | 1 | 4.5 | 4.5 | 81.8 |
| | | 2331.838 | 1 | 4.5 | 4.5 | 86.4 |
| | | 2340.981 | 1 | 4.5 | 4.5 | 90.9 |
| | | 2613.821 | 1 | 4.5 | 4.5 | 95.5 |
| | | 2640.746 | 1 | 4.5 | 4.5 | 100.0 |
| | | Total | 22 | 100.0 | 100.0 | |

| UCH-L1 (pg/mL) | | | | | | |
|---|---|---|---|---|---|---|
| Brain Imaging Result | | | Frequency | Percent | Valid Percent | Cumulative Percent |
| Normal | Valid | 7.119 | 1 | 1.6 | 1.6 | 1.6 |
| | | 8.237 | 1 | 1.6 | 1.6 | 3.3 |
| | | 9.860 | 1 | 1.6 | 1.6 | 4.9 |
| | | 10.236 | 1 | 1.6 | 1.6 | 6.6 |
| | | 10.376 | 1 | 1.6 | 1.6 | 8.2 |
| | | 16.049 | 1 | 1.6 | 1.6 | 9.8 |
| | | 16.674 | 1 | 1.6 | 1.6 | 11.5 |
| | | 17.360 | 1 | 1.6 | 1.6 | 13.1 |
| | | 17.762 | 1 | 1.6 | 1.6 | 14.8 |
| | | 20.162 | 1 | 1.6 | 1.6 | 16.4 |
| | | 20.315 | 1 | 1.6 | 1.6 | 18.0 |
| | | 21.441 | 1 | 1.6 | 1.6 | 19.7 |
| | | 22.706 | 1 | 1.6 | 1.6 | 21.3 |
| | | 23.859 | 1 | 1.6 | 1.6 | 23.0 |
| | | 23.982 | 1 | 1.6 | 1.6 | 24.6 |
| | | 24.056 | 1 | 1.6 | 1.6 | 26.2 |
| | | 24.390 | 1 | 1.6 | 1.6 | 27.9 |
| | | 25.743 | 1 | 1.6 | 1.6 | 29.5 |
| | | 26.802 | 1 | 1.6 | 1.6 | 31.1 |
| | | 27.986 | 1 | 1.6 | 1.6 | 32.8 |
| | | 29.421 | 1 | 1.6 | 1.6 | 34.4 |
| | | 30.515 | 1 | 1.6 | 1.6 | 36.1 |
| | | 30.670 | 1 | 1.6 | 1.6 | 37.7 |
| | | 30.721 | 1 | 1.6 | 1.6 | 39.3 |
| | | 31.590 | 1 | 1.6 | 1.6 | 41.0 |
| | | 31.717 | 1 | 1.6 | 1.6 | 42.6 |
| | | 35.917 | 1 | 1.6 | 1.6 | 44.3 |
| | | 37.743 | 1 | 1.6 | 1.6 | 45.9 |
| | | 38.680 | 1 | 1.6 | 1.6 | 47.5 |
| | | 40.316 | 1 | 1.6 | 1.6 | 49.2 |
| | | 40.877 | 1 | 1.6 | 1.6 | 50.8 |
| | | 42.728 | 1 | 1.6 | 1.6 | 52.5 |
| | | 43.798 | 1 | 1.6 | 1.6 | 54.1 |
| | | 46.483 | 1 | 1.6 | 1.6 | 55.7 |
| | | 47.981 | 1 | 1.6 | 1.6 | 57.4 |
| | | 49.105 | 1 | 1.6 | 1.6 | 59.0 |
| | | 49.901 | 1 | 1.6 | 1.6 | 60.7 |
| | | 50.455 | 1 | 1.6 | 1.6 | 62.3 |
| | | 57.073 | 1 | 1.6 | 1.6 | 63.9 |
| | | 59.750 | 1 | 1.6 | 1.6 | 65.6 |
| | | 60.608 | 1 | 1.6 | 1.6 | 67.2 |
| | | 65.819 | 1 | 1.6 | 1.6 | 68.9 |
| | | 65.977 | 1 | 1.6 | 1.6 | 70.5 |
| | | 67.890 | 1 | 1.6 | 1.6 | 72.1 |
| | | 72.476 | 1 | 1.6 | 1.6 | 73.8 |
| | | 74.218 | 1 | 1.6 | 1.6 | 75.4 |

| UCH-L1 (pg/mL) | | | | | | |
|---|---|---|---|---|---|---|
| Brain Imaging Result | | | Frequency | Percent | Valid Percent | Cumulative Percent |
| | | 83.035 | 1 | 1.6 | 1.6 | 77.0 |
| | | 83.933 | 1 | 1.6 | 1.6 | 78.7 |
| | | 96.265 | 1 | 1.6 | 1.6 | 80.3 |
| | | 102.990 | 1 | 1.6 | 1.6 | 82.0 |
| | | 103.772 | 1 | 1.6 | 1.6 | 83.6 |
| | | 104.450 | 1 | 1.6 | 1.6 | 85.2 |
| | | 107.582 | 1 | 1.6 | 1.6 | 86.9 |
| | | 116.109 | 1 | 1.6 | 1.6 | 88.5 |
| | | 127.456 | 1 | 1.6 | 1.6 | 90.2 |
| | | 127.998 | 1 | 1.6 | 1.6 | 91.8 |
| | | 133.234 | 1 | 1.6 | 1.6 | 93.4 |
| | | 143.043 | 1 | 1.6 | 1.6 | 95.1 |
| | | 183.169 | 1 | 1.6 | 1.6 | 96.7 |
| | | 187.730 | 1 | 1.6 | 1.6 | 98.4 |
| | | 197.634 | 1 | 1.6 | 1.6 | 100.0 |
| | | Total | 61 | 100.0 | 100.0 | |
| Abnormal | Valid | 15.073 | 1 | 4.5 | 4.5 | 4.5 |
| | | 17.039 | 1 | 4.5 | 4.5 | 9.1 |
| | | 18.268 | 1 | 4.5 | 4.5 | 13.6 |
| | | 23.281 | 1 | 4.5 | 4.5 | 18.2 |
| | | 29.540 | 1 | 4.5 | 4.5 | 22.7 |
| | | 30.386 | 1 | 4.5 | 4.5 | 27.3 |
| | | 42.706 | 1 | 4.5 | 4.5 | 31.8 |
| | | 48.201 | 1 | 4.5 | 4.5 | 36.4 |
| | | 49.188 | 1 | 4.5 | 4.5 | 40.9 |
| | | 58.248 | 1 | 4.5 | 4.5 | 45.5 |
| | | 63.553 | 1 | 4.5 | 4.5 | 50.0 |
| | | 83.872 | 1 | 4.5 | 4.5 | 54.5 |
| | | 89.672 | 1 | 4.5 | 4.5 | 59.1 |
| | | 92.724 | 1 | 4.5 | 4.5 | 63.6 |
| | | 100.462 | 1 | 4.5 | 4.5 | 68.2 |
| | | 110.735 | 1 | 4.5 | 4.5 | 72.7 |
| | | 114.594 | 1 | 4.5 | 4.5 | 77.3 |
| | | 135.899 | 1 | 4.5 | 4.5 | 81.8 |
| | | 138.220 | 1 | 4.5 | 4.5 | 86.4 |
| | | 151.345 | 1 | 4.5 | 4.5 | 90.9 |
| | | 386.304 | 1 | 4.5 | 4.5 | 95.5 |
| | | 713.444 | 1 | 4.5 | 4.5 | 100.0 |
| | | Total | 22 | 100.0 | 100.0 | |

| NF-L (pg/mL) | | | | | | |
|---|---|---|---|---|---|---|
| Brain Imaging Result | | | Frequency | Percent | Valid Percent | Cumulative Percent |
| Normal | Valid | 9.055 | 1 | 1.6 | 1.6 | 1.6 |
| | | 9.632 | 1 | 1.6 | 1.6 | 3.3 |
| | | 10.392 | 1 | 1.6 | 1.6 | 4.9 |
| | | 11.916 | 1 | 1.6 | 1.6 | 6.6 |
| | | 12.708 | 1 | 1.6 | 1.6 | 8.2 |
| | | 14.843 | 1 | 1.6 | 1.6 | 9.8 |
| | | 14.976 | 1 | 1.6 | 1.6 | 11.5 |
| | | 15.103 | 1 | 1.6 | 1.6 | 13.1 |
| | | 15.132 | 1 | 1.6 | 1.6 | 14.8 |
| | | 15.252 | 1 | 1.6 | 1.6 | 16.4 |
| | | 17.566 | 1 | 1.6 | 1.6 | 18.0 |
| | | 17.919 | 1 | 1.6 | 1.6 | 19.7 |
| | | 18.001 | 1 | 1.6 | 1.6 | 21.3 |
| | | 18.281 | 1 | 1.6 | 1.6 | 23.0 |
| | | 18.851 | 1 | 1.6 | 1.6 | 24.6 |
| | | 19.801 | 1 | 1.6 | 1.6 | 26.2 |
| | | 20.051 | 1 | 1.6 | 1.6 | 27.9 |
| | | 20.353 | 1 | 1.6 | 1.6 | 29.5 |
| | | 20.806 | 1 | 1.6 | 1.6 | 31.1 |
| | | 20.950 | 1 | 1.6 | 1.6 | 32.8 |
| | | 21.813 | 1 | 1.6 | 1.6 | 34.4 |
| | | 22.368 | 1 | 1.6 | 1.6 | 36.1 |
| | | 22.439 | 1 | 1.6 | 1.6 | 37.7 |
| | | 22.874 | 1 | 1.6 | 1.6 | 39.3 |
| | | 23.078 | 1 | 1.6 | 1.6 | 41.0 |
| | | 24.739 | 1 | 1.6 | 1.6 | 42.6 |
| | | 25.452 | 1 | 1.6 | 1.6 | 44.3 |
| | | 25.808 | 1 | 1.6 | 1.6 | 45.9 |
| | | 26.332 | 1 | 1.6 | 1.6 | 47.5 |
| | | 26.716 | 1 | 1.6 | 1.6 | 49.2 |
| | | 29.610 | 1 | 1.6 | 1.6 | 50.8 |
| | | 30.608 | 1 | 1.6 | 1.6 | 52.5 |
| | | 31.041 | 1 | 1.6 | 1.6 | 54.1 |
| | | 31.277 | 1 | 1.6 | 1.6 | 55.7 |
| | | 31.575 | 1 | 1.6 | 1.6 | 57.4 |
| | | 31.925 | 1 | 1.6 | 1.6 | 59.0 |
| | | 32.702 | 1 | 1.6 | 1.6 | 60.7 |
| | | 36.157 | 1 | 1.6 | 1.6 | 62.3 |
| | | 39.659 | 1 | 1.6 | 1.6 | 63.9 |
| | | 40.205 | 1 | 1.6 | 1.6 | 65.6 |
| | | 44.632 | 1 | 1.6 | 1.6 | 67.2 |
| | | 45.553 | 1 | 1.6 | 1.6 | 68.9 |
| | | 46.989 | 1 | 1.6 | 1.6 | 70.5 |
| | | 47.272 | 1 | 1.6 | 1.6 | 72.1 |
| | | 49.223 | 1 | 1.6 | 1.6 | 73.8 |
| | | 49.444 | 1 | 1.6 | 1.6 | 75.4 |

| NF-L (pg/mL) | | | | | | |
|---|---|---|---|---|---|---|
| Brain Imaging Result | | | Frequency | Percent | Valid Percent | Cumulative Percent |
| | | 53.901 | 1 | 1.6 | 1.6 | 77.0 |
| | | 55.782 | 1 | 1.6 | 1.6 | 78.7 |
| | | 68.159 | 1 | 1.6 | 1.6 | 80.3 |
| | | 69.423 | 1 | 1.6 | 1.6 | 82.0 |
| | | 85.437 | 1 | 1.6 | 1.6 | 83.6 |
| | | 86.047 | 1 | 1.6 | 1.6 | 85.2 |
| | | 86.380 | 1 | 1.6 | 1.6 | 86.9 |
| | | 90.539 | 1 | 1.6 | 1.6 | 88.5 |
| | | 93.206 | 1 | 1.6 | 1.6 | 90.2 |
| | | 99.755 | 1 | 1.6 | 1.6 | 91.8 |
| | | 99.772 | 1 | 1.6 | 1.6 | 93.4 |
| | | 133.355 | 1 | 1.6 | 1.6 | 95.1 |
| | | 135.883 | 1 | 1.6 | 1.6 | 96.7 |
| | | 142.353 | 1 | 1.6 | 1.6 | 98.4 |
| | | 237.303 | 1 | 1.6 | 1.6 | 100.0 |
| | | Total | 61 | 100.0 | 100.0 | |
| Abnormal | Valid | 17.451 | 1 | 4.5 | 4.5 | 4.5 |
| | | 18.326 | 1 | 4.5 | 4.5 | 9.1 |
| | | 19.128 | 1 | 4.5 | 4.5 | 13.6 |
| | | 19.463 | 1 | 4.5 | 4.5 | 18.2 |
| | | 19.594 | 1 | 4.5 | 4.5 | 22.7 |
| | | 19.913 | 1 | 4.5 | 4.5 | 27.3 |
| | | 21.106 | 1 | 4.5 | 4.5 | 31.8 |
| | | 26.612 | 1 | 4.5 | 4.5 | 36.4 |
| | | 27.766 | 1 | 4.5 | 4.5 | 40.9 |
| | | 29.823 | 1 | 4.5 | 4.5 | 45.5 |
| | | 33.862 | 1 | 4.5 | 4.5 | 50.0 |
| | | 35.572 | 1 | 4.5 | 4.5 | 54.5 |
| | | 35.672 | 1 | 4.5 | 4.5 | 59.1 |
| | | 36.954 | 1 | 4.5 | 4.5 | 63.6 |
| | | 38.454 | 1 | 4.5 | 4.5 | 68.2 |
| | | 38.783 | 1 | 4.5 | 4.5 | 72.7 |
| | | 60.286 | 1 | 4.5 | 4.5 | 77.3 |
| | | 62.243 | 1 | 4.5 | 4.5 | 81.8 |
| | | 73.089 | 1 | 4.5 | 4.5 | 86.4 |
| | | 74.804 | 1 | 4.5 | 4.5 | 90.9 |
| | | 90.163 | 1 | 4.5 | 4.5 | 95.5 |
| | | 284.068 | 1 | 4.5 | 4.5 | 100.0 |
| | | Total | 22 | 100.0 | 100.0 | |

| Tau (pg/mL) | | | | | | |
|---|---|---|---|---|---|---|
| Brain Imaging Result | | | Frequency | Percent | Valid Percent | Cumulative Percent |
| Normal | Valid | .171 | 1 | 1.6 | 1.6 | 1.6 |
| | | .533 | 1 | 1.6 | 1.6 | 3.3 |
| | | .627 | 1 | 1.6 | 1.6 | 4.9 |
| | | .721 | 1 | 1.6 | 1.6 | 6.6 |
| | | .814 | 1 | 1.6 | 1.6 | 8.2 |
| | | .826 | 1 | 1.6 | 1.6 | 9.8 |
| | | .846 | 1 | 1.6 | 1.6 | 11.5 |
| | | .894 | 1 | 1.6 | 1.6 | 13.1 |
| | | .911 | 1 | 1.6 | 1.6 | 14.8 |
| | | .919 | 1 | 1.6 | 1.6 | 16.4 |
| | | .969 | 1 | 1.6 | 1.6 | 18.0 |
| | | 1.004 | 1 | 1.6 | 1.6 | 19.7 |
| | | 1.004 | 1 | 1.6 | 1.6 | 21.3 |
| | | 1.074 | 1 | 1.6 | 1.6 | 23.0 |
| | | 1.176 | 1 | 1.6 | 1.6 | 24.6 |
| | | 1.203 | 1 | 1.6 | 1.6 | 26.2 |
| | | 1.207 | 1 | 1.6 | 1.6 | 27.9 |
| | | 1.234 | 1 | 1.6 | 1.6 | 29.5 |
| | | 1.287 | 1 | 1.6 | 1.6 | 31.1 |
| | | 1.312 | 1 | 1.6 | 1.6 | 32.8 |
| | | 1.321 | 1 | 1.6 | 1.6 | 34.4 |
| | | 1.329 | 1 | 1.6 | 1.6 | 36.1 |
| | | 1.395 | 1 | 1.6 | 1.6 | 37.7 |
| | | 1.399 | 1 | 1.6 | 1.6 | 39.3 |
| | | 1.531 | 1 | 1.6 | 1.6 | 41.0 |
| | | 1.598 | 1 | 1.6 | 1.6 | 42.6 |
| | | 1.661 | 1 | 1.6 | 1.6 | 44.3 |
| | | 1.684 | 1 | 1.6 | 1.6 | 45.9 |
| | | 1.706 | 1 | 1.6 | 1.6 | 47.5 |
| | | 1.766 | 1 | 1.6 | 1.6 | 49.2 |
| | | 1.811 | 1 | 1.6 | 1.6 | 50.8 |
| | | 1.820 | 1 | 1.6 | 1.6 | 52.5 |
| | | 1.909 | 1 | 1.6 | 1.6 | 54.1 |
| | | 1.912 | 1 | 1.6 | 1.6 | 55.7 |
| | | 1.948 | 1 | 1.6 | 1.6 | 57.4 |
| | | 2.013 | 1 | 1.6 | 1.6 | 59.0 |
| | | 2.019 | 1 | 1.6 | 1.6 | 60.7 |
| | | 2.104 | 1 | 1.6 | 1.6 | 62.3 |
| | | 2.178 | 1 | 1.6 | 1.6 | 63.9 |
| | | 2.232 | 1 | 1.6 | 1.6 | 65.6 |
| | | 2.233 | 1 | 1.6 | 1.6 | 67.2 |
| | | 2.305 | 1 | 1.6 | 1.6 | 68.9 |
| | | 2.416 | 1 | 1.6 | 1.6 | 70.5 |
| | | 2.439 | 1 | 1.6 | 1.6 | 72.1 |
| | | 2.500 | 1 | 1.6 | 1.6 | 73.8 |
| | | 2.577 | 1 | 1.6 | 1.6 | 75.4 |

| Tau (pg/mL) | | | | | | |
|---|---|---|---|---|---|---|
| Brain Imaging Result | | | Frequency | Percent | Valid Percent | Cumulative Percent |
| | | 2.720 | 1 | 1.6 | 1.6 | 77.0 |
| | | 2.789 | 1 | 1.6 | 1.6 | 78.7 |
| | | 2.802 | 1 | 1.6 | 1.6 | 80.3 |
| | | 2.880 | 1 | 1.6 | 1.6 | 82.0 |
| | | 3.226 | 1 | 1.6 | 1.6 | 83.6 |
| | | 3.306 | 1 | 1.6 | 1.6 | 85.2 |
| | | 3.594 | 1 | 1.6 | 1.6 | 86.9 |
| | | 3.866 | 1 | 1.6 | 1.6 | 88.5 |
| | | 4.534 | 1 | 1.6 | 1.6 | 90.2 |
| | | 4.794 | 1 | 1.6 | 1.6 | 91.8 |
| | | 5.216 | 1 | 1.6 | 1.6 | 93.4 |
| | | 5.261 | 1 | 1.6 | 1.6 | 95.1 |
| | | 5.401 | 1 | 1.6 | 1.6 | 96.7 |
| | | 7.286 | 1 | 1.6 | 1.6 | 98.4 |
| | | 9.448 | 1 | 1.6 | 1.6 | 100.0 |
| | | Total | 61 | 100.0 | 100.0 | |
| Abnormal | Valid | .563 | 1 | 4.5 | 4.5 | 4.5 |
| | | .708 | 1 | 4.5 | 4.5 | 9.1 |
| | | .767 | 1 | 4.5 | 4.5 | 13.6 |
| | | .774 | 1 | 4.5 | 4.5 | 18.2 |
| | | .890 | 1 | 4.5 | 4.5 | 22.7 |
| | | .924 | 1 | 4.5 | 4.5 | 27.3 |
| | | 1.021 | 1 | 4.5 | 4.5 | 31.8 |
| | | 1.133 | 1 | 4.5 | 4.5 | 36.4 |
| | | 1.189 | 1 | 4.5 | 4.5 | 40.9 |
| | | 1.208 | 1 | 4.5 | 4.5 | 45.5 |
| | | 1.745 | 1 | 4.5 | 4.5 | 50.0 |
| | | 1.953 | 1 | 4.5 | 4.5 | 54.5 |
| | | 1.968 | 1 | 4.5 | 4.5 | 59.1 |
| | | 2.036 | 1 | 4.5 | 4.5 | 63.6 |
| | | 2.385 | 1 | 4.5 | 4.5 | 68.2 |
| | | 3.300 | 1 | 4.5 | 4.5 | 72.7 |
| | | 3.366 | 1 | 4.5 | 4.5 | 77.3 |
| | | 3.481 | 1 | 4.5 | 4.5 | 81.8 |
| | | 3.503 | 1 | 4.5 | 4.5 | 86.4 |
| | | 6.328 | 1 | 4.5 | 4.5 | 90.9 |
| | | 6.424 | 1 | 4.5 | 4.5 | 95.5 |
| | | 12.200 | 1 | 4.5 | 4.5 | 100.0 |
| | | Total | 22 | 100.0 | 100.0 | |

**Raw Data for Each Subject**

| | Brain Imaging Result | Age | Time between Injury and blood sampling (hours) | Time between injury and head CT (hours) | GFAP (pg/mL) | UCH-L1 (pg/mL) | NF-L (pg/mL) | Tau (pg/mL) |
|---|---|---|---|---|---|---|---|---|
| 1 | Normal | 60.0 | 3.7 | 5.2 | 94.557 | 7.119 | 9.632 | .171 |
| 2 | Normal | 61.0 | 3.5 | 6.8 | 434.286 | 107.582 | 9.055 | 1.531 |
| 3 | Normal | 63.0 | 3.7 | 4.2 | 193.154 | 17.360 | 133.355 | .627 |
| 4 | Normal | 64.0 | 8.4 | 8.8 | 292.916 | 9.860 | 22.368 | 1.399 |
| 5 | Normal | 65.0 | 2.4 | 4.7 | 135.817 | 24.390 | 14.976 | 1.004 |
| 6 | Normal | 66.0 | 1.9 | 5.8 | 262.556 | 25.743 | 15.252 | .919 |
| 7 | Normal | 66.0 | 3.3 | 6.3 | 92.736 | 8.237 | 44.632 | 1.909 |
| 8 | Normal | 66.0 | .8 | 1.4 | 243.461 | 183.169 | 24.739 | 3.866 |
| 9 | Normal | 67.0 | 1.3 | 1.9 | 105.283 | 60.608 | 20.051 | .826 |
| 10 | Normal | 68.0 | .8 | 4.1 | 170.991 | 20.162 | 31.041 | .533 |
| 11 | Normal | 68.0 | 2.1 | 2.3 | 146.852 | 35.917 | 12.708 | 2.789 |
| 12 | Normal | 68.0 | 2.8 | 5.2 | 322.340 | 17.762 | 30.608 | 2.720 |
| 13 | Normal | 69.0 | 1.3 | 3.3 | 245.478 | 42.728 | 31.575 | 1.766 |
| 14 | Normal | 69.0 | 3.1 | 3.1 | 218.441 | 10.236 | 15.132 | .846 |
| 15 | Normal | 70.0 | 5.1 | 6.2 | 98.566 | 127.998 | 10.392 | .814 |
| 16 | Normal | 70.0 | 3.4 | 5.7 | 190.954 | 24.056 | 14.843 | 1.074 |
| 17 | Normal | 70.0 | 1.4 | 2.3 | 145.567 | 83.035 | 25.808 | 1.811 |
| 18 | Normal | 71.0 | 2.4 | 4.5 | 90.265 | 22.706 | 22.874 | 1.598 |
| 19 | Normal | 72.0 | 1.6 | 1.7 | 169.771 | 72.476 | 15.103 | 3.594 |
| 20 | Normal | 72.0 | 4.3 | 5.6 | 219.909 | 16.674 | 49.444 | 2.233 |
| 21 | Normal | 73.0 | 9.1 | 10.0 | 308.590 | 10.376 | 11.916 | 2.178 |
| 22 | Normal | 74.0 | 1.2 | 2.4 | 567.810 | 83.933 | 32.702 | 3.226 |
| 23 | Normal | 75.0 | 1.4 | 1.8 | 166.290 | 65.977 | 17.919 | 2.019 |
| 24 | Normal | 75.0 | 3.6 | 5.0 | 504.937 | 26.802 | 23.078 | 3.306 |
| 25 | Normal | 76.0 | 1.2 | 1.5 | 529.588 | 21.441 | 22.439 | 1.004 |
| 26 | Normal | 77.0 | 2.1 | 2.2 | 210.562 | 31.717 | 18.851 | 1.912 |
| 27 | Normal | 77.0 | 4.7 | 4.8 | 192.155 | 47.981 | 20.806 | 1.661 |
| 28 | Normal | 77.0 | 1.7 | 3.1 | 477.162 | 23.982 | 18.281 | 1.287 |
| 29 | Normal | 77.0 | 5.7 | 5.7 | 233.167 | 96.265 | 39.659 | 1.207 |
| 30 | Normal | 78.0 | 5.2 | 6.0 | 260.781 | 67.890 | 19.801 | 2.802 |
| 31 | Normal | 78.0 | 6.1 | 5.5 | 106.699 | 74.218 | 31.925 | 1.948 |
| 32 | Normal | 78.0 | 5.4 | 5.6 | 323.188 | 37.743 | 21.813 | 5.261 |
| 33 | Normal | 78.0 | 1.4 | 1.4 | 422.222 | 104.450 | 90.539 | 5.216 |
| 34 | Normal | 80.0 | 1.0 | 4.6 | 263.371 | 29.421 | 17.566 | 1.176 |
| 35 | Normal | 81.0 | 3.1 | 3.2 | 215.777 | 103.772 | 25.452 | 1.706 |
| 36 | Normal | 81.0 | 11.7 | 16.4 | 627.396 | 30.670 | 86.047 | .911 |
| 37 | Normal | 81.0 | 3.8 | 6.7 | 788.310 | 43.798 | 68.159 | 9.448 |
| 38 | Normal | 82.0 | 8.8 | 10.3 | 433.279 | 30.515 | 53.901 | 2.500 |
| 39 | Normal | 83.0 | 1.8 | 5.3 | 306.735 | 40.877 | 36.157 | 2.416 |
| 40 | Normal | 83.0 | 3.4 | 6.1 | 330.417 | 16.049 | 47.272 | 1.234 |
| 41 | Normal | 83.0 | 1.1 | 3.0 | 332.453 | 65.819 | 99.755 | 2.013 |
| 42 | Normal | 83.0 | 1.3 | 1.6 | 294.218 | 27.986 | 86.380 | 1.312 |
| 43 | Normal | 84.0 | 3.3 | 3.1 | 387.479 | 23.859 | 26.716 | 1.820 |

| | Brain Imaging Result | Age | Time between Injury and blood sampling (hours) | Time between injury and head CT (hours) | GFAP (pg/mL) | UCH-L1 (pg/mL) | NF-L (pg/mL) | Tau (pg/mL) |
|---|---|---|---|---|---|---|---|---|
| 44 | Normal | 84.0 | 4.0 | 17.9 | 235.187 | 20.315 | 26.332 | 2.104 |
| 45 | Normal | 85.0 | 4.1 | 14.3 | 532.299 | 143.043 | 20.353 | .969 |
| 46 | Normal | 86.0 | 2.9 | 1.6 | 87.404 | 102.990 | 18.001 | 1.321 |
| 47 | Normal | 86.0 | 9.0 | 9.1 | 501.701 | 57.073 | 46.989 | 1.329 |
| 48 | Normal | 88.0 | 2.9 | 4.5 | 853.919 | 197.634 | 93.206 | 2.577 |
| 49 | Normal | 88.0 | 1.8 | 3.4 | 403.082 | 127.456 | 40.205 | 7.286 |
| 50 | Normal | 88.0 | 4.7 | 4.9 | 560.016 | 38.680 | 29.610 | 5.401 |
| 51 | Normal | 88.0 | 5.2 | 5.4 | 753.972 | 59.750 | 69.423 | 2.232 |
| 52 | Normal | 89.0 | 1.5 | 1.6 | 434.825 | 49.901 | 49.223 | 2.439 |
| 53 | Normal | 89.0 | 3.6 | 3.8 | 3382.409 | 116.109 | 142.353 | 2.880 |
| 54 | Normal | 90.0 | 4.0 | 6.0 | 274.270 | 31.590 | 55.782 | 1.203 |
| 55 | Normal | 90.0 | 1.8 | 2.1 | 460.618 | 187.730 | 135.883 | .721 |
| 56 | Normal | 91.0 | 2.3 | 2.4 | 221.865 | 30.721 | 20.950 | 1.395 |
| 57 | Normal | 93.0 | 5.3 | 5.6 | 424.888 | 133.234 | 99.772 | 4.534 |
| 58 | Normal | 93.0 | 1.9 | 9.9 | 557.109 | 49.105 | 237.303 | 4.794 |
| 59 | Normal | 94.0 | 2.3 | 2.7 | 267.663 | 40.316 | 45.553 | 2.305 |
| 60 | Normal | 94.0 | 2.7 | 4.6 | 272.902 | 46.483 | 31.277 | .894 |
| 61 | Normal | 100.0 | 4.9 | 4.7 | 610.401 | 50.455 | 85.437 | 1.684 |
| 62 | Abnormal | 61.0 | 5.1 | 4.8 | 846.644 | 48.201 | 73.089 | 1.968 |
| 63 | Abnormal | 68.0 | 3.3 | 3.4 | 2057.879 | 151.345 | 19.913 | 3.481 |
| 64 | Abnormal | 71.0 | 8.7 | 10.0 | 533.145 | 30.386 | 33.862 | .767 |
| 65 | Abnormal | 72.0 | 4.3 | 8.3 | 349.994 | 15.073 | 19.463 | .708 |
| 66 | Abnormal | 72.0 | 3.8 | 4.2 | 2212.887 | 100.462 | 19.594 | 1.021 |
| 67 | Abnormal | 73.0 | 4.6 | 6.0 | 195.592 | 713.444 | 90.163 | 6.424 |
| 68 | Abnormal | 73.0 | 1.3 | 1.4 | 386.933 | 23.281 | 74.804 | 1.745 |
| 69 | Abnormal | 76.0 | 1.3 | 2.0 | 657.108 | 89.672 | 26.612 | 2.385 |
| 70 | Abnormal | 77.0 | 2.9 | 2.7 | 1191.149 | 63.553 | 29.823 | 3.300 |
| 71 | Abnormal | 80.0 | 1.3 | 2.6 | 2613.821 | 138.220 | 21.106 | 3.366 |
| 72 | Abnormal | 82.0 | 3.9 | 6.3 | 2331.838 | 58.248 | 18.326 | .890 |
| 73 | Abnormal | 83.0 | 5.3 | 13.6 | 1382.719 | 110.735 | 19.128 | 1.953 |
| 74 | Abnormal | 85.0 | 2.6 | 2.3 | 729.044 | 18.268 | 35.572 | 1.133 |
| 75 | Abnormal | 86.0 | 2.9 | 3.6 | 354.694 | 17.039 | 35.672 | .924 |
| 76 | Abnormal | 87.0 | 2.5 | 2.5 | 323.110 | 29.540 | 17.451 | .774 |
| 77 | Abnormal | 88.0 | 4.5 | 5.4 | 492.604 | 49.188 | 38.454 | .563 |
| 78 | Abnormal | 89.0 | 4.8 | 6.8 | 1372.245 | 135.899 | 36.954 | 1.208 |
| 79 | Abnormal | 89.0 | 8.3 | 7.6 | 2640.746 | 92.724 | 60.286 | 3.503 |
| 80 | Abnormal | 89.0 | 2.9 | 3.0 | 1064.121 | 114.594 | 284.068 | 12.200 |
| 81 | Abnormal | 92.0 | 1.4 | 2.8 | 188.588 | 42.706 | 27.766 | 6.328 |
| 82 | Abnormal | 95.0 | 5.9 | 5.9 | 2340.981 | 83.872 | 62.243 | 2.036 |
| 83 | Abnormal | 96.0 | 1.3 | 4.3 | 114.444 | 386.304 | 38.783 | 1.189 |
